# Frequency reconfigurable PIN diode-based Reuleaux-triangle-shaped monopole antenna for UWB/Ku band applications

**DOI:** 10.1038/s41598-025-91108-7

**Published:** 2025-02-24

**Authors:** Sena Esen Bayer Keskin, Slawomir Koziel, Stanislaw Szczepanski

**Affiliations:** 1 Department of Electrical and Electronics Engineering, Kırklareli University, 39000 Kırklareli, Turkey; 2 Engineering Optimization and Modeling Center, Reykjavik University, 101 Reykjavík, Iceland; 3 Faculty of Electronics, Telecommunications and Informatics, Gdansk University of Technology, 80-233 Gdańsk, Poland

**Keywords:** Frequency reconfigurable antenna, Monopole antenna, UWB band, Ku band, PIN diode, Electrical and electronic engineering, Engineering

## Abstract

This paper presents a frequency reconfigurable monopole antenna developed for UWB/Ku band applications. The design employs a microstrip-fed Reuleaux-triangle-shaped patch with a defected ground structure. The antenna exhibits a wide operating bandwidth achieved due to rectangular slits integrated into the Reuleaux-triangle patch. Meanwhile, adding rectangular slots in the ground plane improves the return loss level. Frequency reconfigurability is obtained by utilizing PIN diodes to adjust the current distribution, altering the antenna’s electrical length via the capacitive and inductive effects induced by the rings near the feed line. The antenna operates in two distinct frequency bands, 2.68–8.55 GHz and 12.7–15.65 GHZ, contingent upon the PIN-diodes’ ON/OFF states. In the OFF state, the antenna covers the UWB region, in particular, the ISM band (5.8 GHz), WLAN band (5.2 GHz), and lower X-band (8 GHz), exhibiting a 10 dB impedance bandwidth from 2.68 to 8.55 GHz with a maximum gain of 2.36 dBi. In the ON state, the antenna functions in the Ku band (12.7–15.65 GHz) with gains from 2.63 to 3.85 dBi. The antenna’s dynamic switching between UWB and Ku band operations makes it suitable for applications such as satellite communications, health monitoring, 5G, aerospace, and remote sensing.

## Introduction

Nowadays, there is a growing interest in developing antennas capable of operating efficiently across multiple frequency bands. This includes ultra-wideband (UWB) and Ku band applications. UWB provides high data rates and low power consumption and facilitates short-range communication, making it ideal for radar systems, wireless communication, and imaging applications. At the same time, Ku band supports high-frequency, long-range transmissions critical for satellite communications and weather radar systems. UWB and Ku bands enable a broad spectrum of communication technologies, driving the need for antennas that can operate efficiently across these bands. Extensive research has focused on antennas operating within the UWB and Ku bands due to their importance in modern communication systems. The demand for UWB patch antennas has risen since the Federal Communication Commission (FCC) designated the 3.1–10.6 GHz band, prompting techniques like stacking the patch antenna^1^, slot and notch on the patch^2^, use of planar monopole antennas and defective ground structures to improve bandwidth and gain^3,4^.

In practical scenarios, such as critical real-time command and control applications or scenarios with stringent latency requirements, IoT, medical applications such as cancer detection or remote robotic surgery, radar systems, or autonomous vehicle communication, the low-latency characteristics of the UWB spectrum become of paramount importance^5–12^. On the other hand, in satellite communication systems, the demand for high-data-rate transfers, such as those required for transmitting large volumes of data or high-resolution imagery, can be optimally addressed by the Ku band. Consequently, recent research has shifted toward developing compact UWB patch antennas for higher frequency ranges (> 10 GHz) due to congestion in lower frequencies, with a focus on Ku/K band applications (12–20 GHz)^3,13–15^. Some studies present antennas effectively covering both UWB and Ku bands^16–21^. For instance, in a study by El-Hakim et al., a microstrip antenna with dimensions of 46 × 38 × 1.6 mm^3^ was proposed, achieving peak gain values of 2.8 dBi, 3.8 dBi, and 4.7 dBi at resonant frequencies of 2.45 GHz, 6 GHz, and 14 GHz, respectively, covering both UWB and Ku bands^16^. In another study conducted by Ansal et al. a compact co-planar waveguide-fed antenna was designed for UWB and Ku band applications, covering 3.11–18.03 GHz with stable gain and radiation patterns, utilizing circular incisions to achieve wideband and Ku band performance^17^.

The frequency reconfiguration is an important functionality required nowadays in antennas, enabling them to switch between different bands for various applications and reducing the need for multiple antennas^22^. Notably, growing research focuses on antennas capable of dynamically switching between multi-band configurations in real time. These frequency reconfigurable antennas are important in wireless communication systems due to their adaptability across various frequency bands. Numerous studies have explored and advanced the development of reconfigurable antennas^23–28^. Frequency reconfigurability is often achieved through switches such as RF MEMS, PIN diodes, varactor diodes, etc.^29^. MEMS switches are favored in wireless applications for their excellent isolation and linearity. However, a notable limitation is their requirement for high DC control voltages^30^. PIN and Varactor diodes are the most commonly used switches due to their fast switching capabilities and compact size^31–33^. The switching speed of a PIN diode is in the range of 1–100 nsec^32^. Reconfigurable antennas with PIN diodes provide dynamic switching, while those with varactors adjust capacitance by varying the biasing voltage^34^. Some studies achieve re-configurable band rejection through the use of PIN diodes^35–37^. For instance, Mayuri et al*.* proposed a compact reconfigurable dual-band notched UWB monopole antenna with a wide impedance bandwidth of 8.33 GHz, covering 3.17–11.61 GHz, providing reconfigurable band notch in WiMAX and WLAN bands^35^. Similarly, Sharma et al*.* presented a compact monopole antenna with reconfigurable band-notch characteristics, capable of dynamically rejecting WiMAX, WLAN, and downlink satellite bands while covering Bluetooth, LTE, UWB, X band, and Ku band frequencies through the use of PIN diodes^36^. In 2019, Beigi et al*.* developed a 4-mode frequency reconfigurable antenna that operates based on the modes of PIN diodes, covering the 3.5–20 GHz range, including both UWB and Ku bands, or exhibiting band rejection at 5–6.2 GHz, 9.5–10.2 GHz, and 5–6.3 GHz, 9.5–11 GHz^37^. However, these antennas cannot reconfigure frequencies between the UWB and Ku bands. These studies are limited to reconfigurability within either the UWB or Ku bands individually, or they encompass both bands without offering dynamic frequency switching. Consequently, while numerous studies have investigated UWB and Ku band antennas, many lack the capability for dynamic switching between these bands^38^. This limitation restricts their adaptability in multi-band applications. Recent advancements in wireless communication systems have focused increasingly on developing antennas capable of operating across multiple frequency bands.

This study introduces an innovative structure of a frequency reconfigurable Reuleaux-triangle-shaped monopole (R-TSM) antenna that can dynamically switch between UWB and Ku bands and allows for rejecting the X band, adapting to a wide range of communication needs. The wide operating bandwidth is realized by incorporating rectangular slits integrated into the radiating patch, whereas implementing rectangular slots into the ground plane enhances impedance matching. Frequency reconfigurability is obtained using the PIN diodes that alter the antenna’s electrical length via the capacitive and inductive effects of the rings positioned near the feed line. The R-TSM antenna’s dual-band operation and dynamic switching capability between UWB and Ku bands enable versatile applications, addressing high data rate transmission needs in the Ku band and low-latency communication requirements in the UWB spectrum within a single antenna system. Additionally, the antenna’s rejection of the higher frequencies in the X band proves beneficial in radar systems, especially in scenarios where interference from such signals needs to be minimized. This could include weather radar or ground-based surveillance radar applications. Competitive functionality and performance of the proposed design were demonstrated through extensive benchmarking against state-of-the-art designs reported in the recent literature.

## Materials and methods

**Fig. 1:**
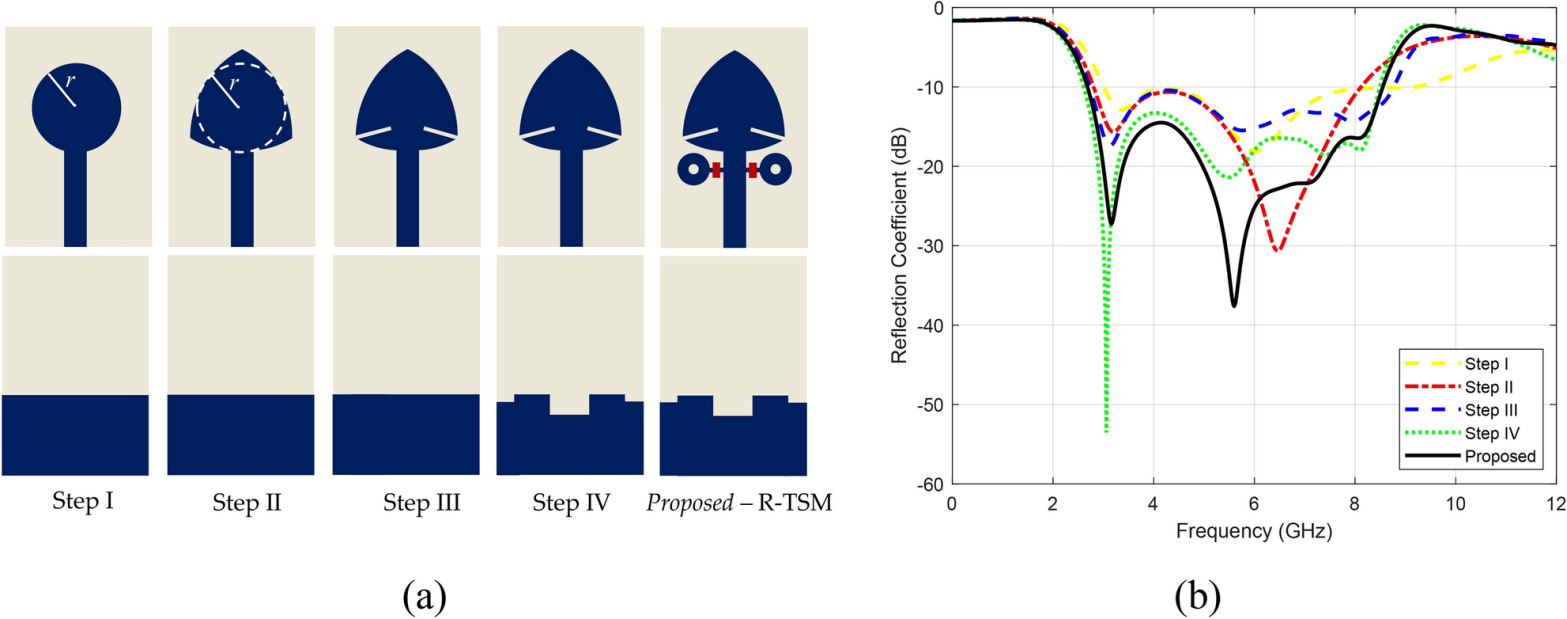
The proposed R-TSM antenna: (**a**) design steps, (**b**) corresponding reflection coefficients.

Planar monopole antennas are often favored for UWB applications due to their structural simplicity, ease of fabrication, wideband properties, and the ability to radiate in all directions. These features make them suitable for diverse communication scenarios. Therefore, the development of the proposed antenna starts with a circular monopole patch. It has been shown that monopole antennas with a finite ground plane can support multiple resonant modes instead of a single resonant mode compared to those with a complete ground plane (conventional circular patch antennas)^39^. The overlapping of closely spaced multiple resonance modes can provide wide bandwidth^40^. The circular monopole patch’s radius $R$ as^39^

1 $$R = \frac{{R_{eff} }}{{\sqrt {\left( {1 + \frac{2h}{{\pi \varepsilon_{r} R_{eff} }}\left[ {\ln \left( {\frac{{1.57R_{eff} }}{h}} \right) + 1.78} \right]} \right)} }}$$

where the effective radius $R_{eff}$ is given by

2 $$R_{eff} = \frac{{8.79 \times 10^{9} }}{{f_{r} \sqrt {\varepsilon_{r} } }}$$

here $f_{r}$ is the resonance frequency, $\varepsilon_{r}$ is the dielectric constant and $h$ is the hight of the substrate material, where $h$ must be in centimeters ($cm$). Here, the resonant frequency is for a circular disc antenna etched on a printed-circuit board, where the low-profile radiating element (the circular disc) is separated from the ground plane by a thin layer of dielectric material^41^. Based on these equations, the radius $r$ of the circular monopole patch is selected as 6 mm. The evolution from a circular monopole to a Reuleaux-triangle-shaped antenna aims to enhance the antenna performance, offering advantages like increased compactness, expanded bandwidth, multiband operation potential, and directional characteristics. The antenna’s Reuleaux-triangle shape, also known as a spherical triangle, is formed by drawing arcs from the vertices of an equilateral triangle. Specifically, a circle is drawn from each vertex with a radius equal to the side length of the triangle, where each arc intersects the other two vertices. For a Reuleaux-triangle, despite its rounded, non-circular appearance, the width (the perpendicular distance between two parallel tangent lines) is constant in all directions. This unique shape maintains constant width during rotation, offering beneficial electromagnetic properties for antenna design. The geometry of the Reuleaux-triangle-shaped monopole antenna, illustrated in Fig. 1- Step I, is characterized by the following dimensions: length of 30 mm, width of 20 mm, and a ground plane length of 11 mm. Antenna design steps are illustrated in Fig. 1), where blue indicates the patch surface, and light yellow represents the dielectric. The reflection coefficient values corresponding to these design steps are illustrated in Fig. 1b.

**Fig. 2:**
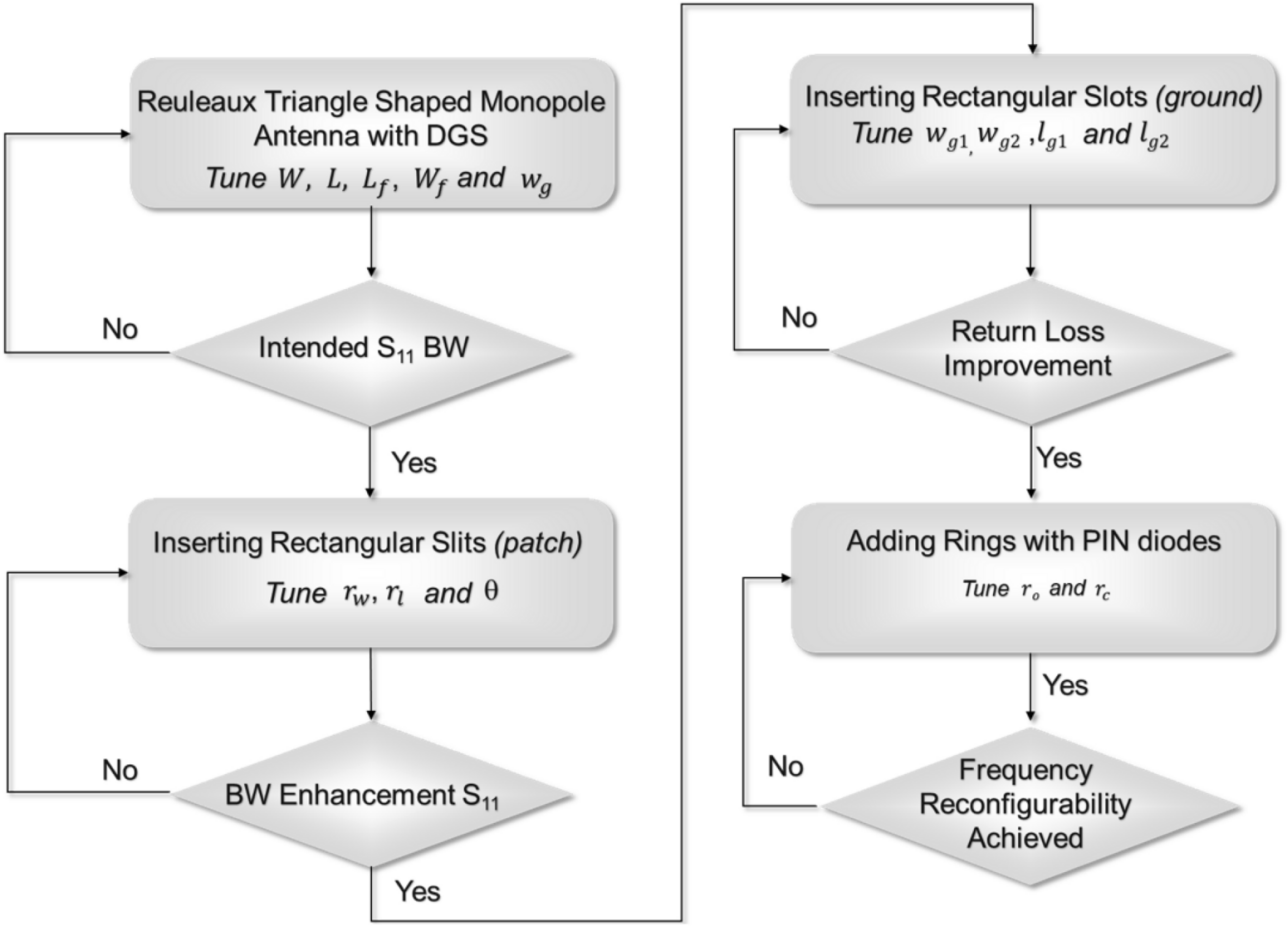
Flowchart of the design steps of the proposed R-TSM antenna.

The design steps of the frequency reconfigurable R-TSM antenna are summarized in the flowchart shown in Fig. 2. Initially, a Reuleaux-triangle-shaped monopole antenna was selected due to its wider bandwidth compared to traditional circular and rectangular monopole antennas. Rectangular slits are etched on the patch, which modify the surface current distribution and create multiple resonances, resulting in bandwidth enhancement. This has been illustrated in Fig. 1, specifically, a comparison between Step II and Step III of antenna geometry development. To further enhance the bandwidth, additional rectangular slots are strategically introduced to the ground plane to optimize impedance matching. These modifications improve the antenna’s ability to cover a broader frequency range, addressing the need for UWB applications. Two rings are added on both sides of the feed line to achieve frequency reconfigurability. The PIN diodes are positioned between these rings and the feed line, with the anode on the side adjacent to the ring. Incorporating PIN diodes enables dynamic adjustment of the operating frequency, allowing the antenna to switch between different frequency bands.

**Fig. 3:**
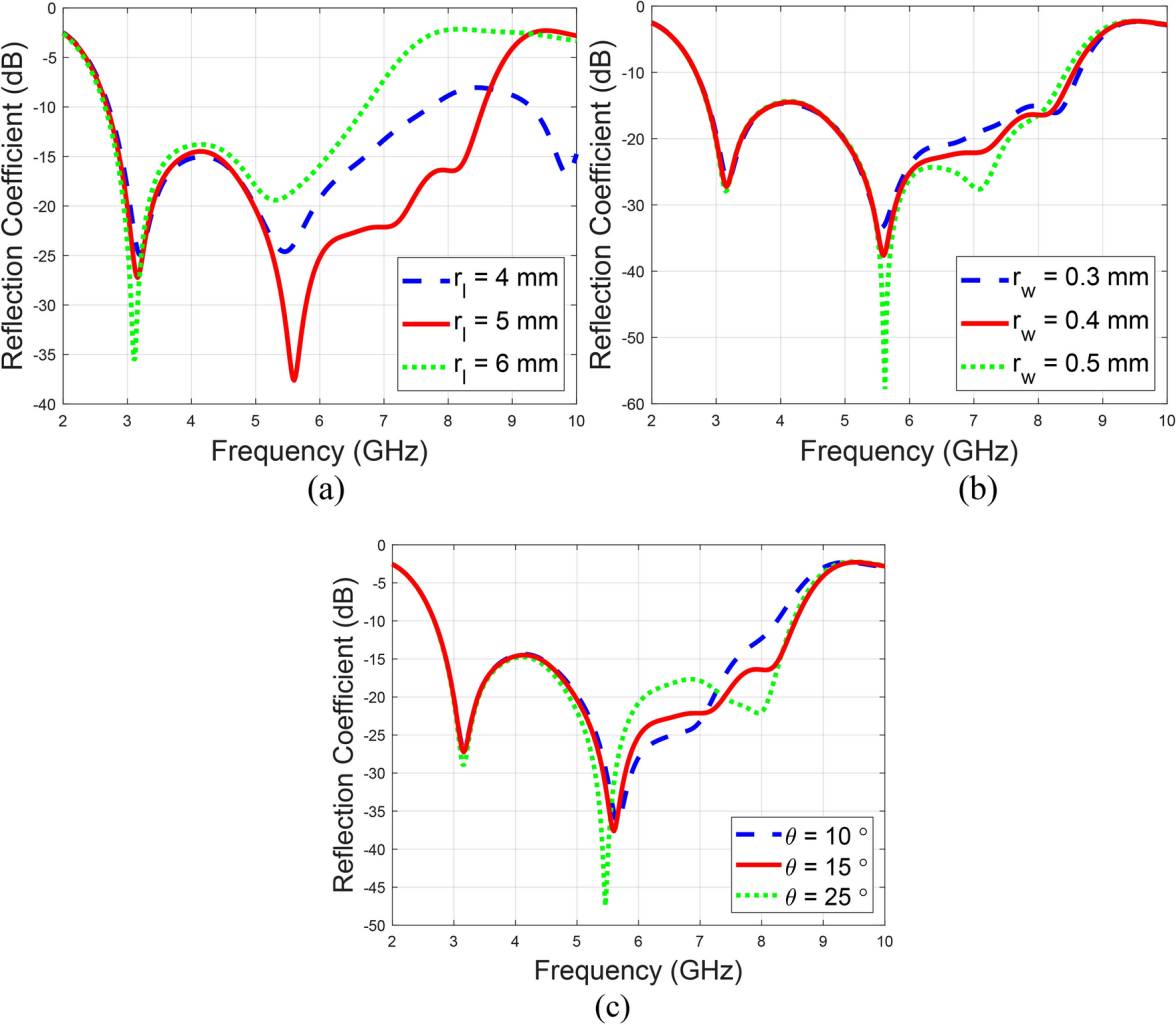
Parametric analysis of the rectangular slit dimensions, (**a**) $r_{l}$, (**b**) $r_{w}$, and (**c**) *q*.

In this study, the width of $r_{w}$, length of $r_{l}$, and angle *q* of a rectangular slit etched at a 15-degree angle on the patch surface of the antenna are optimized to assess their impact on the bandwidth. Among these parameters, $r_{l}$ is identified as the most critical factor influencing bandwidth, with an optimal length of 5 mm. The relevant return reflection characteristics are illustrated in Fig. 3, highlighting the improvement in impedance bandwidth.

**Fig. 4:**
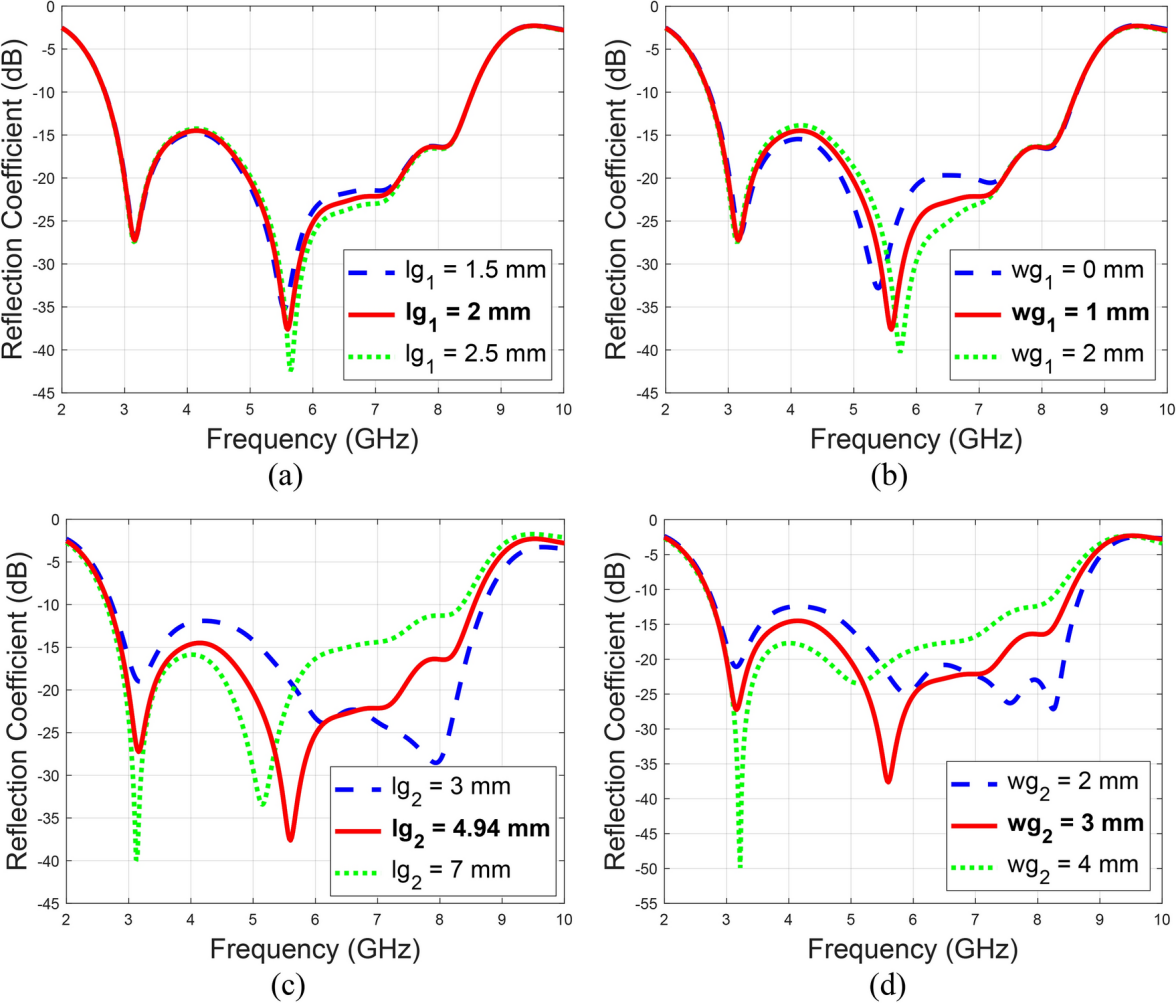
Parametric analysis of the rectangular ground plane slot dimensions, (**a**) $lg_{1}$, (**b**) $wg_{1}$, (**c**) $lg_{2}$, and (**d**) $wg_{2}$.

Figure 4 illustrates the parametric analysis for the rectangular slots placed on the ground plane to optimize the impedance matching. The study includes three slots: one positioned just behind the microstrip feed, one at the left-top edge, and one at the right-top edge of the ground plane. The optimization revealed that the slot behind the microstrip feed significantly impacts the reflection response, with the optimum dimensions being 4.94 mm in length and 3 mm in width. The slots at the edges of the ground plane also affect the return loss, with their optimum dimensions being 2 mm in length and 1 mm in width.

**Fig. 5:**
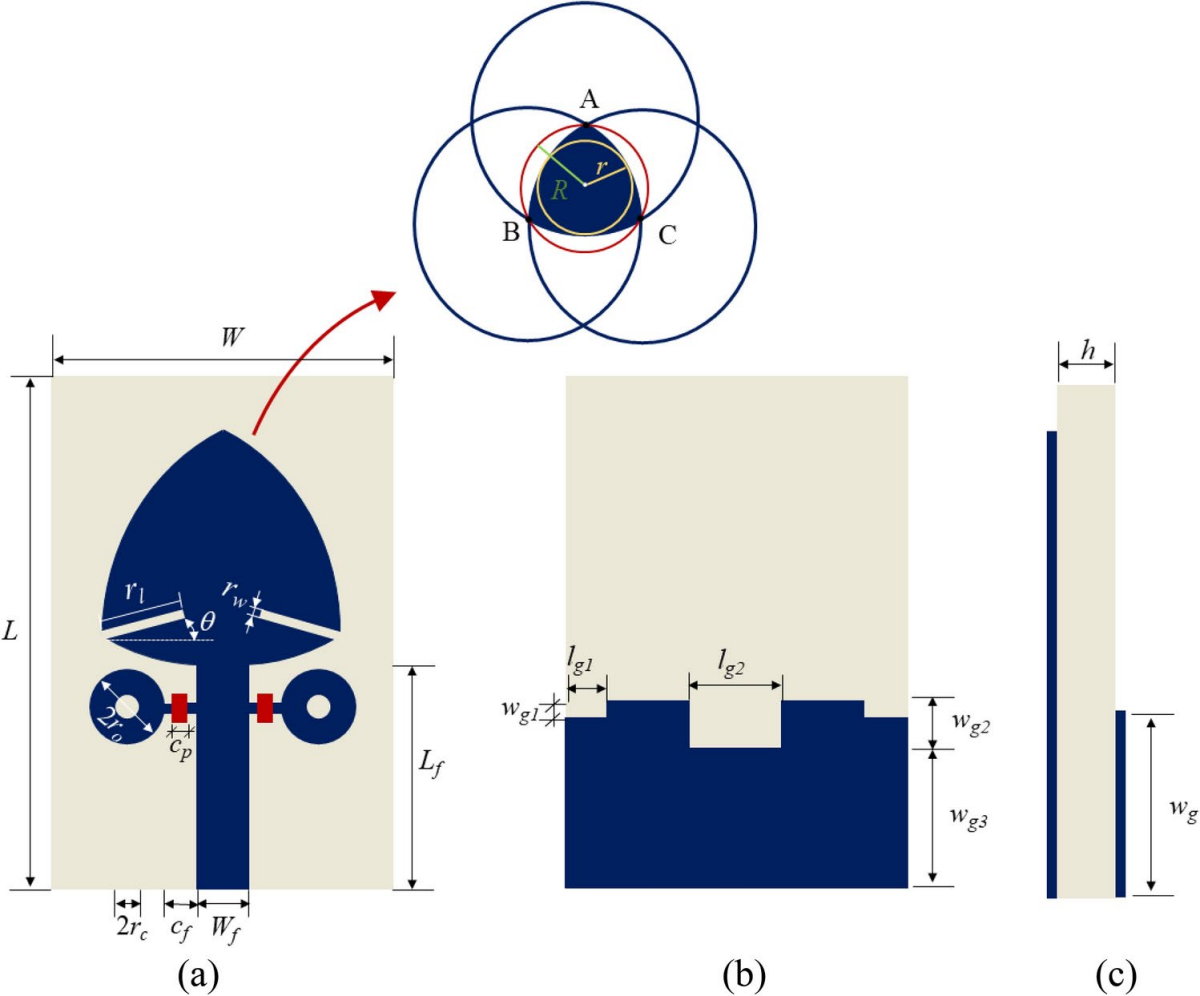
Geometry of R-TSM antenna: (**a**) top view, (**b**) bottom view, (**c**) side view.

**Fig. 6:**
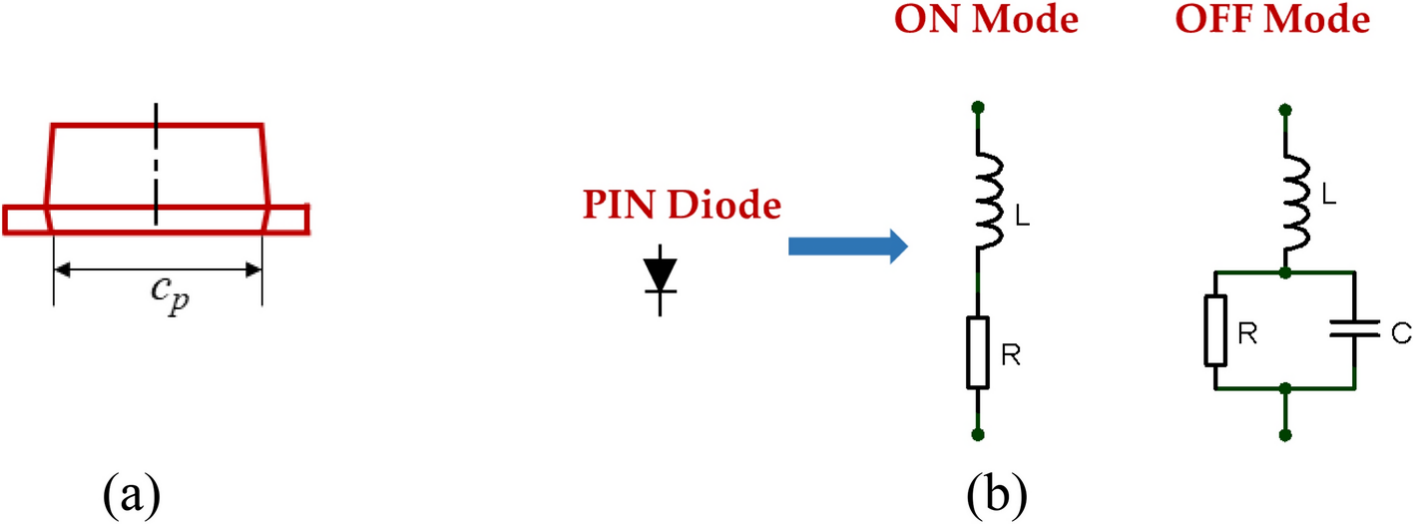
PIN Diodes, (**a**) PIN diode schematic, and (**b**) equivalent circuit model.

Figure 5 provides a detailed dimension labeling of the proposed frequency reconfigurable R-TSM antenna, with red rectangles indicating the locations of the PIN diodes. As previously described, a Reuleaux-triangle is constructed by connecting the vertices of an equilateral triangle using circular arcs, cf. Figure 5. In this diagram, the vertices of the equilateral triangle are labeled A, B, and C. Circles are drawn with centers at these vertices, each having a radius equal to the side length of the triangle, and their intersection creates the Reuleaux triangle. Additionally, two reference circles are depicted: an inner circle with a radius r and an outer circle with a radius *R*. The design begins with a circular monopole patch, with a radius of 6 mm. It evolved into a Reuleaux-triangle-shaped monopole antenna, where the inner circle’s radius is 6 mm to enhance antenna performance. The BAP 51-02 PIN diode manufactured by NXP is chosen to provide frequency tunability to the antenna operating in the UWB/Ku Band due to its advantageous features, such as low capacitance, fast switching speed, and high reverse breakdown voltage. The Pin diode schematic and equivalent circuit model are depicted in Fig. 6.

**Fig. 7:**
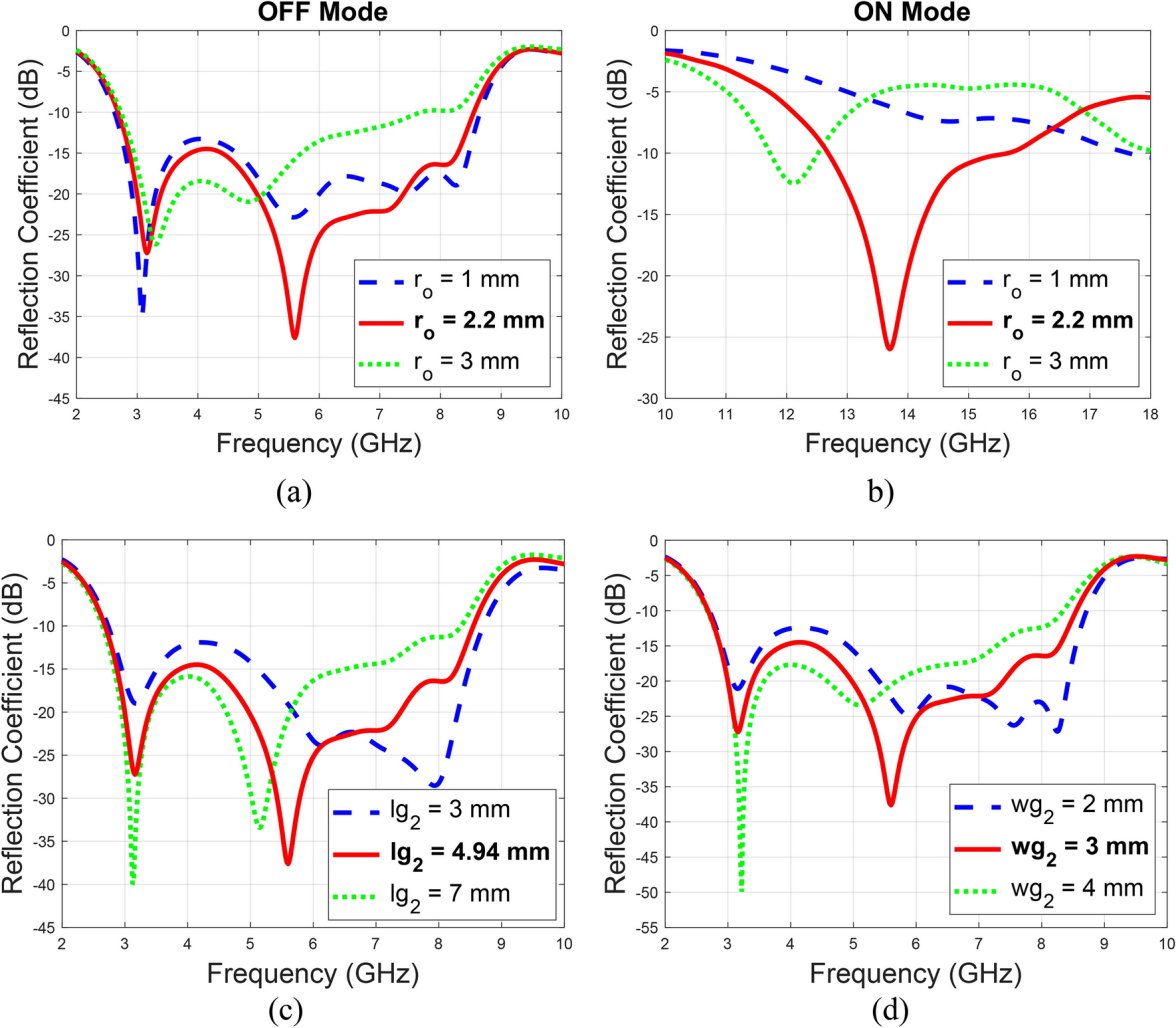
Parametric analysis of the ring diameters for reconfigurability connected with the PIN diodes: (**a**) the effects of the outer ring diameter $r_{o}$ in OFF Mode, (**b**) in ON Mode, (**c**) the effects of the inner ring diameter $r_{c}$ in OFF Mode, (**d**) in ON Mode.

Figure 7 presents the size optimization results for the circular rings used to achieve frequency tunability in the UWB band of the designed antenna. In the considered configuration, the outer radius $r_{o}$ of the circular ring is set to 2.2 mm to facilitate the antenna’s operation in the Ku band when the diode is in the ON state. The inner radius $r_{c}$ is set to 0.7 mm. The dimension optimization of these circular rings for both ON and OFF states of the PIN diode has been meticulously performed. The results demonstrate the practical tuning of the antenna’s frequency response, as illustrated in Fig. 7. This optimization allows for enhanced frequency agility, enabling the antenna to cover the UWB band efficiently while switching to the Ku band as required.

The dimensions of the optimized frequency reconfigurable R-TSM antenna have been provided in Table 1. The relative permittivity of the substrate material is $\varepsilon_{r}$ = 4.4, whereas the tangent loss (tan $\delta )$ is 0.02. The copper thickness of the FR-4 epoxy substrate is 35 µm. The rectangular slits on the patch are tilted at an angle $\theta$ = 15 degrees.

**Table 1:** Dimensions parameters of the frequency reconfigurable R-TSM antenna.

Parameter	Value (mm)	Parameter	Value (mm)	Parameter	Value (mm)
$L$	30	$W$	20	$c_{p}$	1.15
$L_{f}$	13.19	$W_{f}$	2.94	$c_{f}$	2.16
$w_{g1}$	1	$l_{g1}$	2	$r_{c}$	0.7
$w_{g2}$	3	$l_{g2}$	4.94	$r_{o}$	2.2
$w_{g}$	11	$r_{l}$	5	$h$	1.6
$r$	6	$r_{w}$	0.4		

**Fig. 8:**
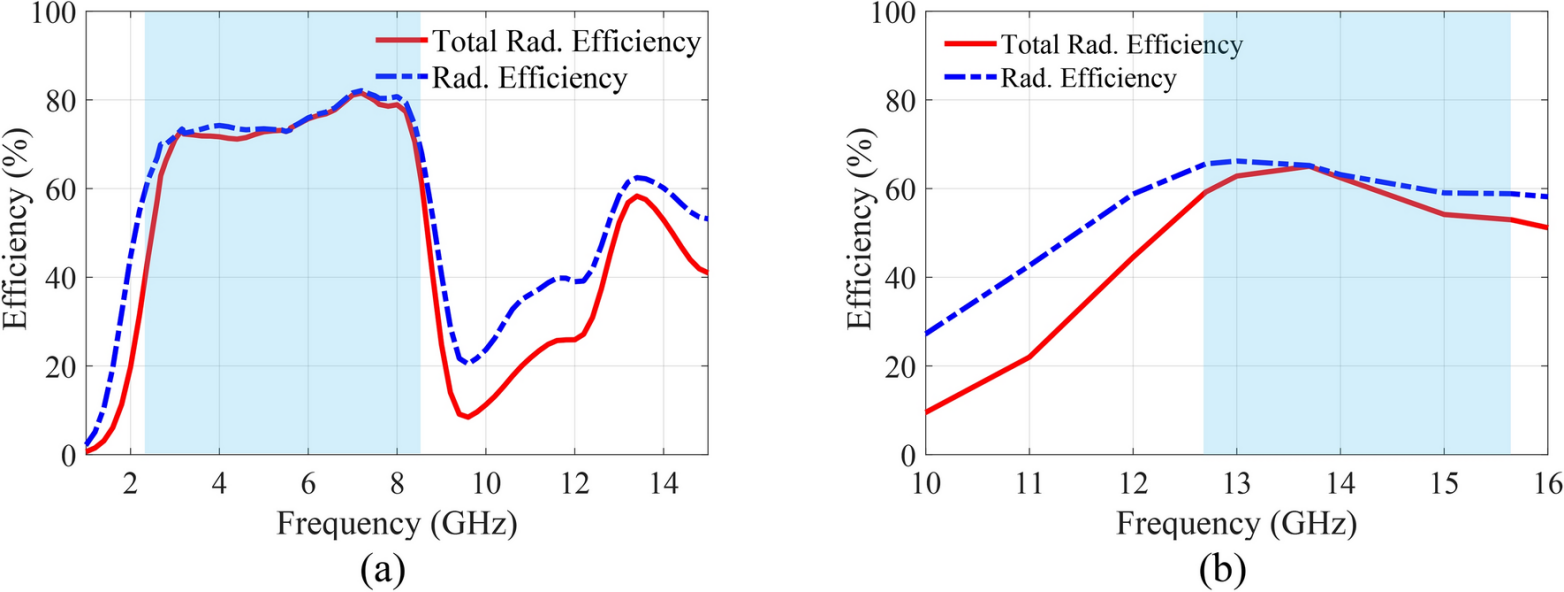
Simulated total radiation efficiency and radiation efficiency of the R-TSM antenna: (**a**) OFF Mode, (**b**) ON Mode.

**Fig. 9.**
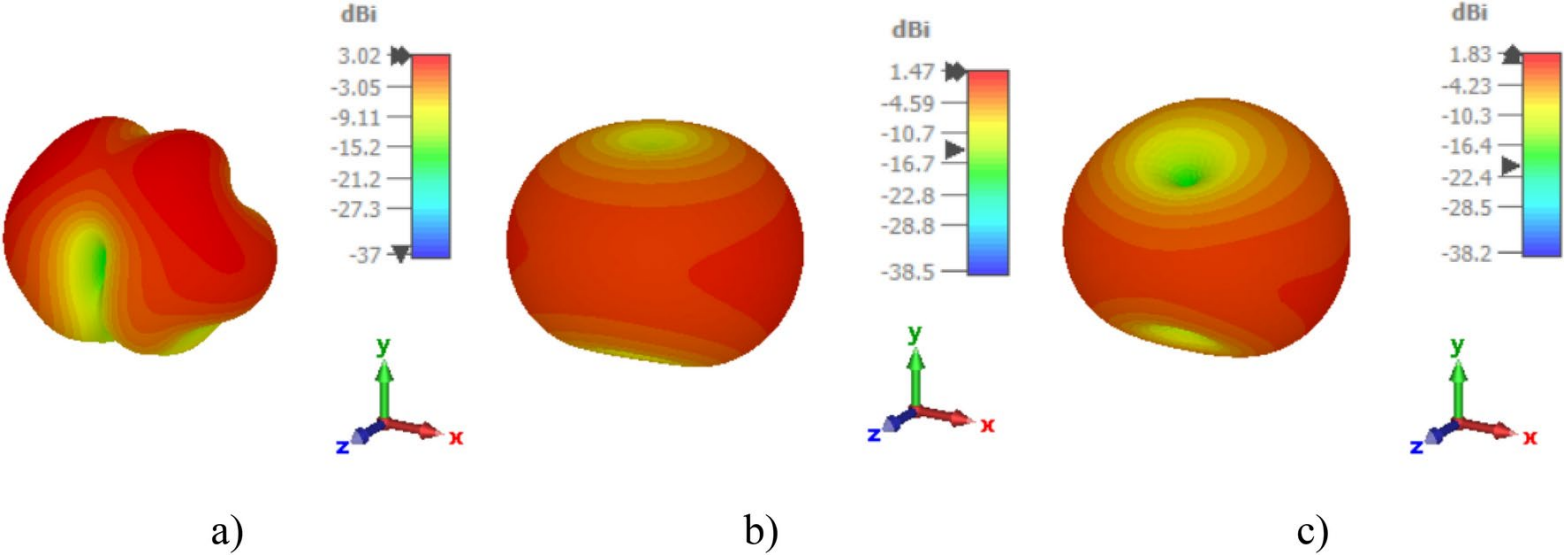
3D gain of the R-TSM antenna: (**a**) at $f$ = 13.7 GHz in the ON mode, (**b**) at $f$ = 3.16 GHz in the OFF mode, (**c**) at $f$ = 5.6 GHz in the OFF mode.

Based on the simulation results, the proposed R-TSM antenna demonstrates practically sufficient radiation efficiency, as depicted in Fig. 8. The radiation efficiency exceeds 60% for both the OFF and ON modes across the operating frequency range. Specifically, a maximum total efficiency of 65% is observed in the ON mode at the resonance frequency of 13.7 GHz, while the OFF mode achieves a peak efficiency of 81.5% at the resonance frequency of 7.2 GHz. The 3D gain patterns of the proposed R-TSM antenna are shown in Fig. 9 for both ON and OFF modes. The patterns are evaluated at the resonance frequency of 13.7 GHz for the ON mode and at 3.16 GHz and 5.6 GHz for the OFF mode.

**Fig. 10:**
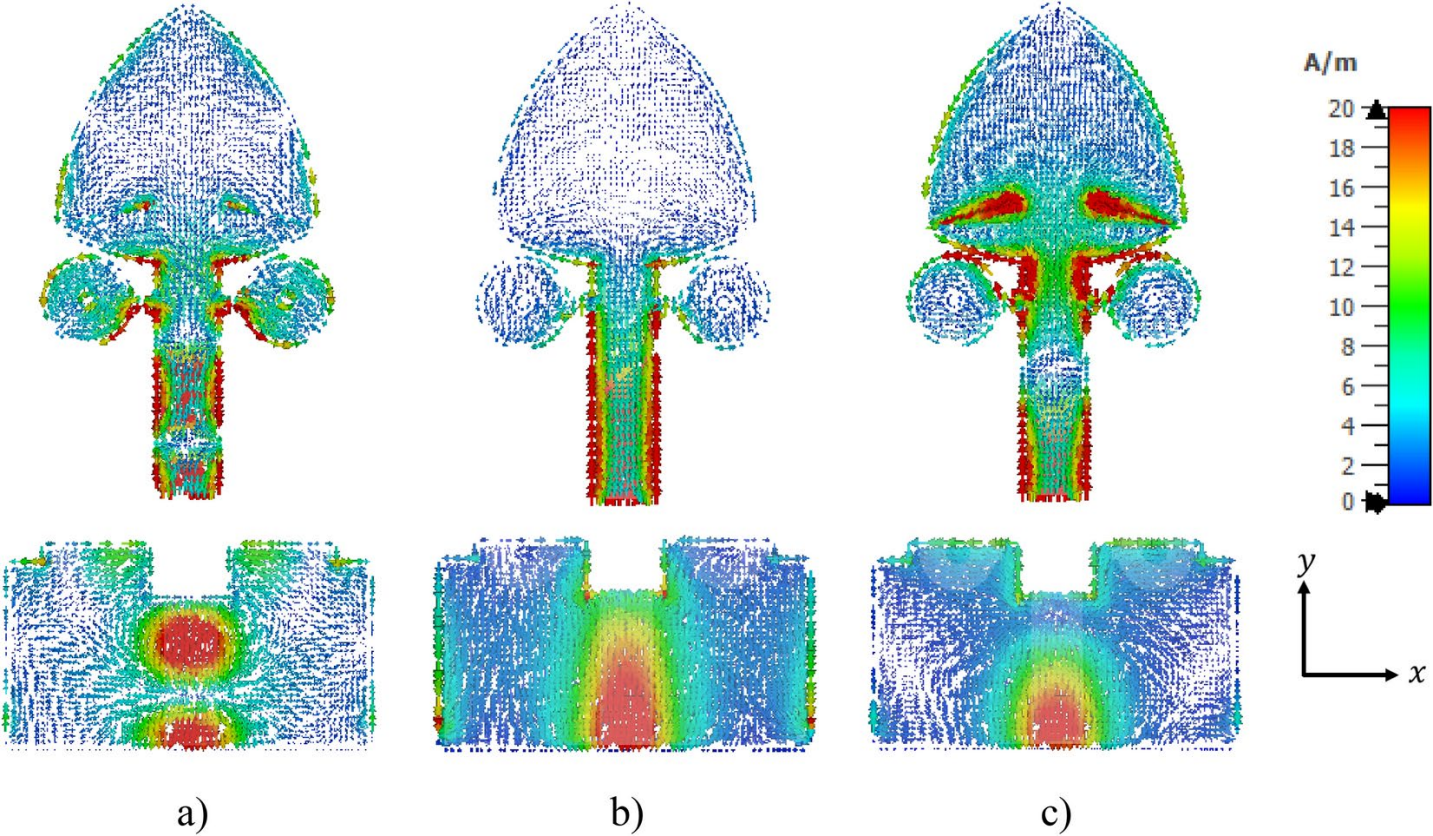
Distribution of surface current for the proposed R-TSM antenna: (**a**) at $f$ = 13.7 GHz in the ON mode, (**b**) at $f$ = 3.16 GHz in the OFF mode, (**c**) at $f$ = 5.6 GHz in the OFF mode.

To further explain the antenna operation in the context of frequency reconfigurability, the simulated surface current distributions at the resonance frequencies of 13.7 GHz in the ON mode, and 3.16 GHz and 5.6 GHz in the OFF mode, are shown in Fig. 10a–c. In the simulations of surface current distribution at the resonant frequencies of 5.6 GHz in the OFF mode and 13.7 GHz in the ON mode, significant surface currents are observed around the circles used for reconfigurability. In contrast, at 3.16 GHz, the current density around these circles is notably lower. Additionally, at 5.6 GHz in OFF mode, the slits on the patch exhibit a high current density, with a slightly lower yet still noticeable current density at 13.7 GHz in ON mode, highlighting the slits’ role in tuning the antenna’s performance at these frequencies. In contrast, at 3.16 GHz, the current density on the slits is minimal, indicating a reduced contribution of the slits to resonance at this lower frequency.

## Results and discussion

**Fig. 11:**
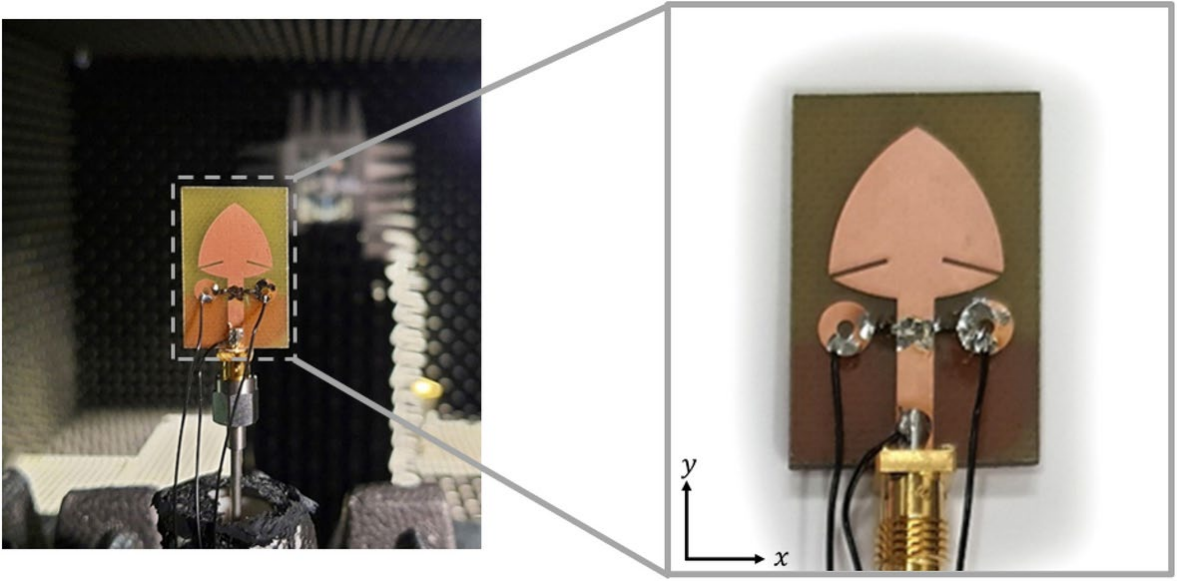
The prototype of the fabricated R-TSM antenna placed within the measurement setup.

**Fig. 12:**
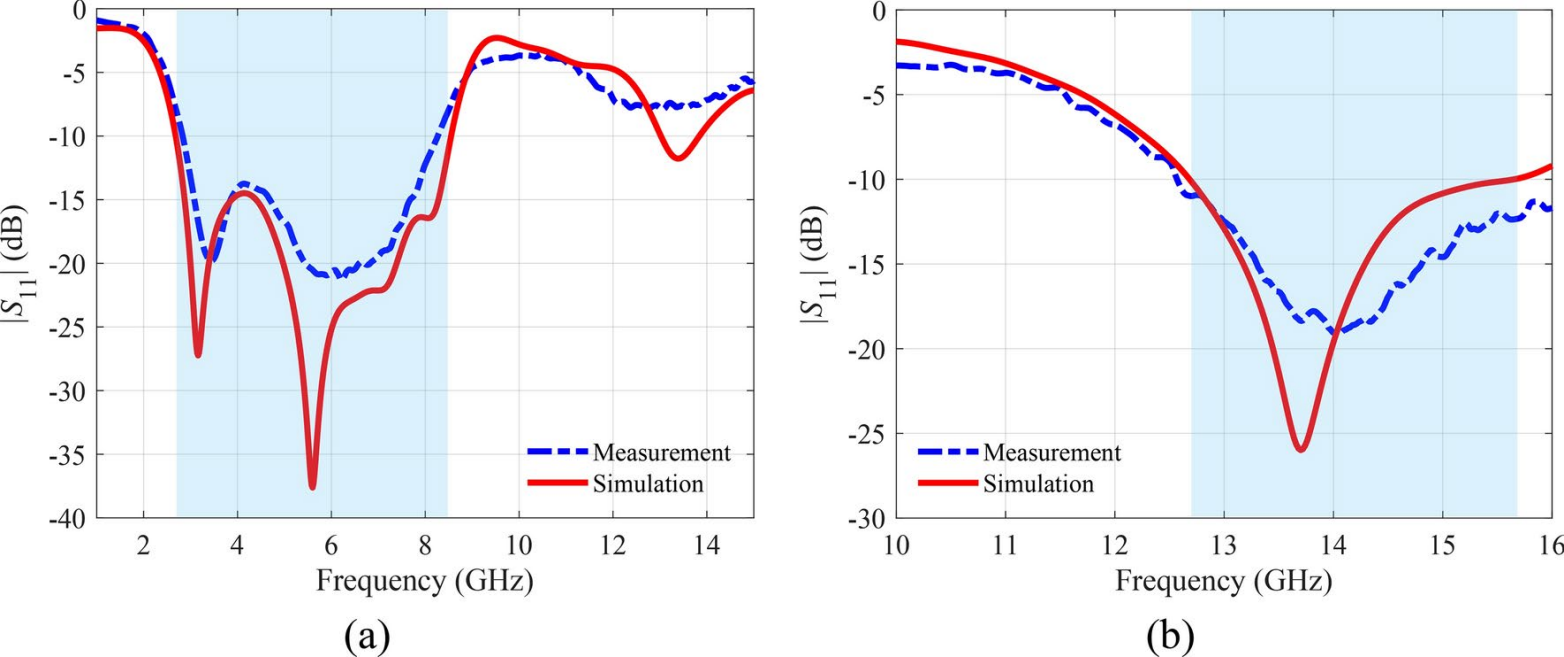
Reflection coefficient values of proposed R-TSM: (**a**) OFF mode, (**b**) ON mode.

For experimental validation, the presented frequency-reconfigurable antenna was fabricated and measured in the anechoic chamber. The photograph of the prototype and the measurement setup are shown in Fig. 11. The two PIN diodes used for dynamical switching are controlled by bias voltages applied across the connecting wires, as seen in the expanded view. The wire soldered to the microstrip feed line is connected to the negative terminal of the DC power supply. In contrast, the remaining wires are connected to the positive terminal of the DC power supply. A regulated DC power supply GW Instek GPS-4303 is utilized to provide the necessary DC bias voltage. The measurements were carried out using 0–40 GHz Anritsu MS4644B vector network analyzer. The comparison between simulation and measurement for the OFF mode is presented in Fig. 12a, whereas the ON mode results are shown in Fig. 12b. Both modes show good agreement between the simulated and measured results. Minor variations, however, can be assumed due to the possible fabrication in-accuracies, impedance mismatch at the soldered SMA connector, and the non-ideal behavior of the PIN diodes used for frequency switching.

**Fig. 13:**
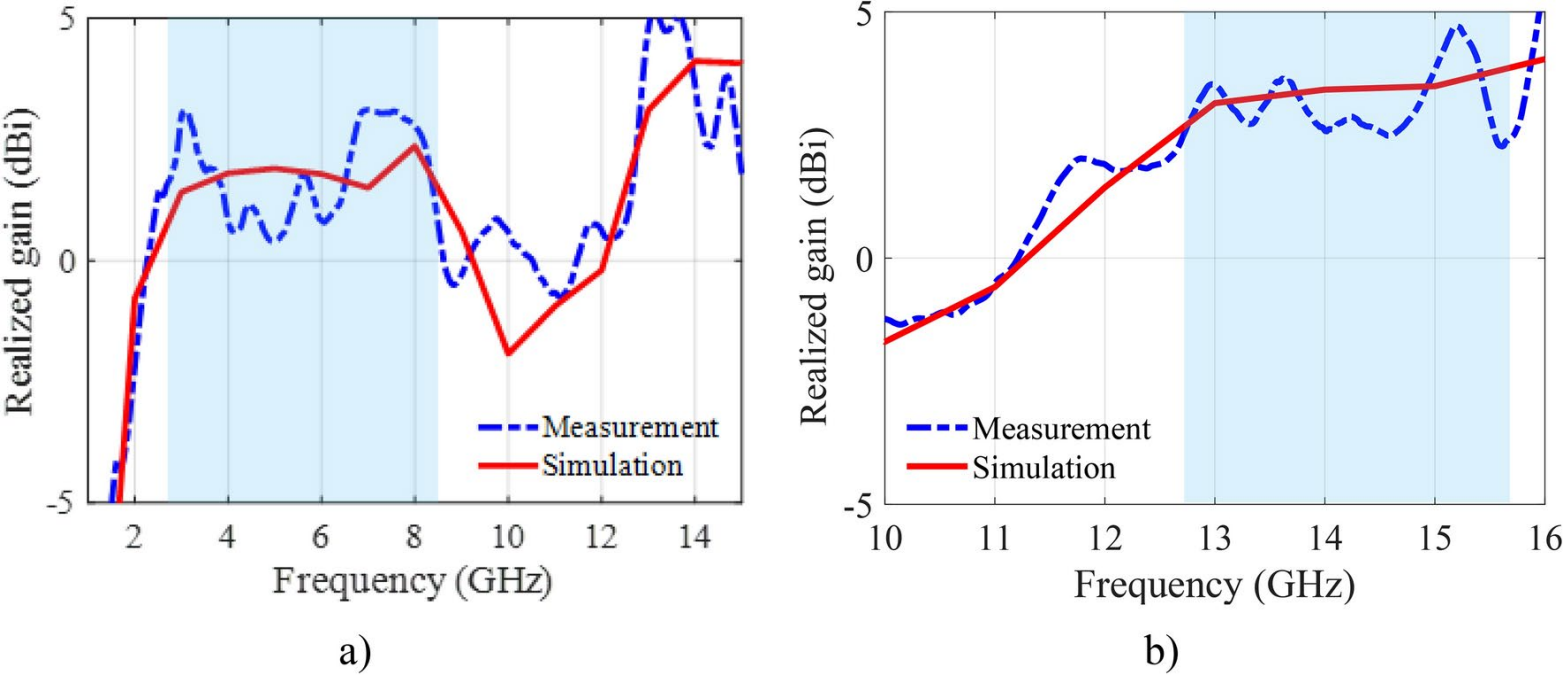
Realized gain: (**a**) OFF mode, (**b**) ON mode.

**Fig. 14:**
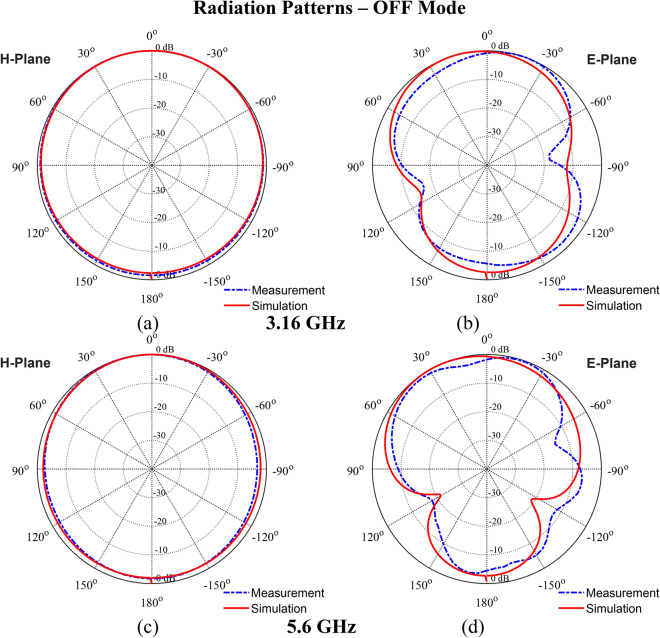
Radiation pattern in OFF Mode at 3.16 GHz: (**a**) H-plane, (**b**) E-plane, and at 5.6 GHz: (**c**) H-plane, (**d**) E-plane.

**Fig. 15:**
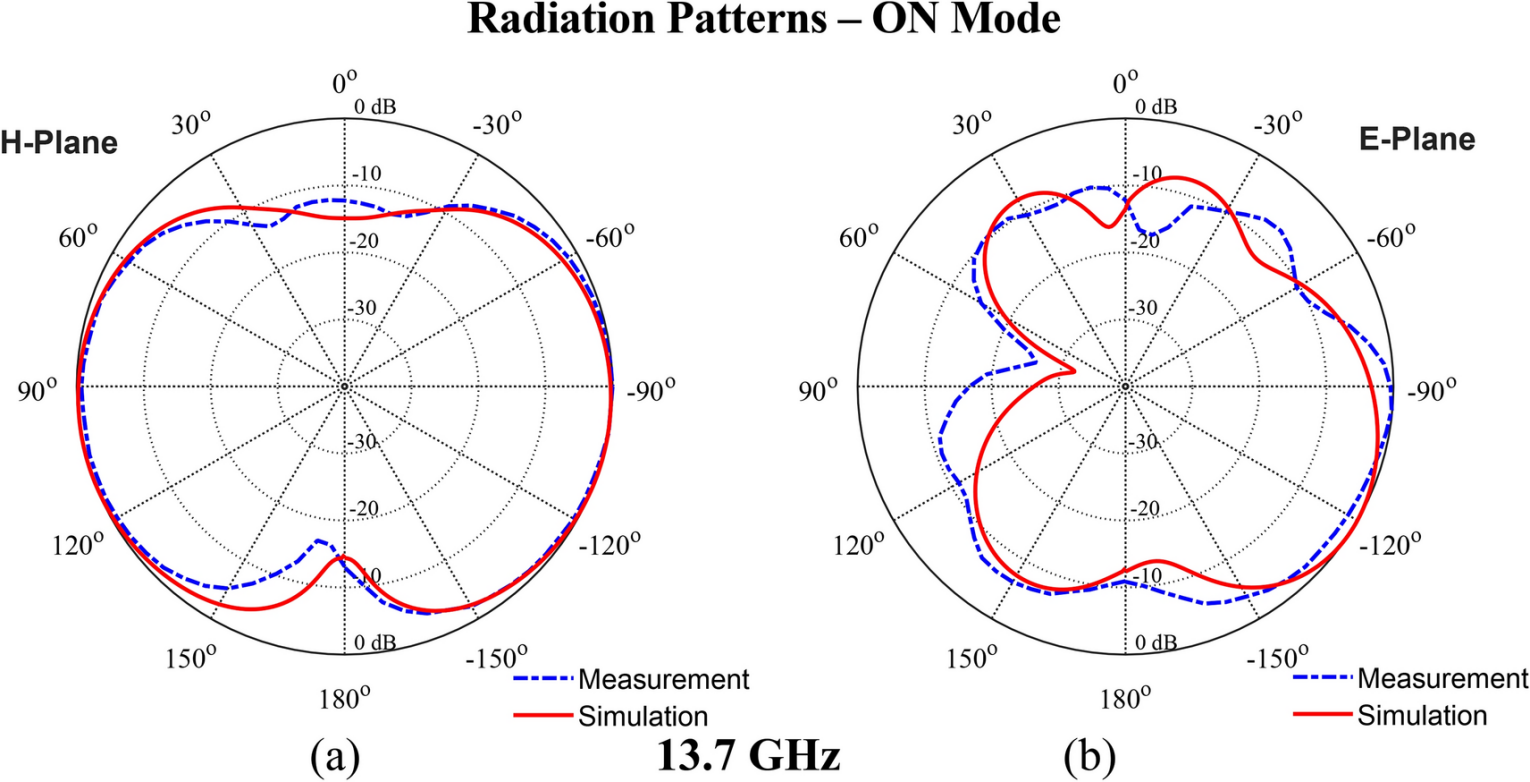
Radiation pattern in ON Mode at 13.7 GHz: (**a**) H-plane, and (**b**) E-plane.

The simulated gain results for the OFF and ON modes are depicted in Fig. 13a,b, respectively. In the OFF mode, the antenna achieves a peak gain of 2.36 dBi. In ON mode, the gain varies between 2.63 and 3.85 dBi, reflecting an increase in performance due to the activation of the PIN diodes. Although the proposed monopole microstrip antenna has lower gain, this is an inherent property of monopole antennas due to their omnidirectional radiation patterns, which prioritize wide coverage rather than high gain, making it suitable for targeted broadband applications such as frequency switching between bands UWB and Ku-band. Furthermore, a close alignment between the simulated and measured gain values in both modes confirms the reliability of the antenna’s reconfigurable design. Figures 14 and 15 present the simulated and measured radiation characteristics of the antenna (both H- and E-plane) in the OFF mode and the ON mode. The patterns are at 3.16 GHz and 5.6 GHz (OFF mode), at 13.7 GHz (ON mode). All radiation patterns exhibit omnidirectional characteristics for each configuration.

### Radiation patterns—OFF mode

### Radiation patterns—ON mode

**Fig. 16:**
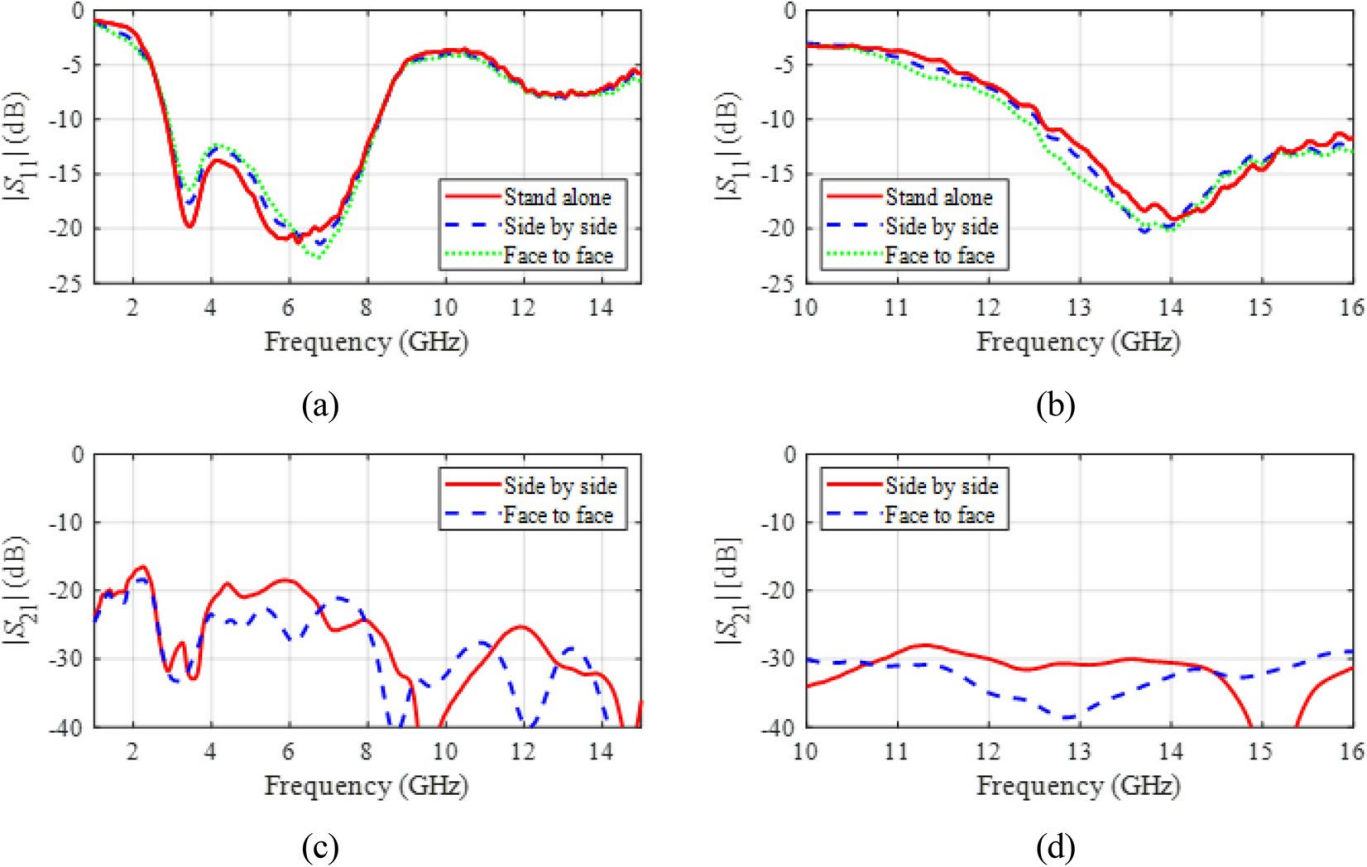
*S*-parameters for MIMO configuration (two copies of the antenna allocated four centimeters between the feed lines): (**a**) reflection responses for the OFF mode, (**b**) reflection responses for the ON mode, (**c**) |*S*_21_| for the OFF mode, (**d**) |*S*_21_| for the ON mode.

Figure 16 shows the measured *S*-parameters for the proposed antenna in MIMO configuration (two copies separated by four centimeters measured between the feedline axes). As observed, the reflection coefficient is insensitive to the presence of another antenna, whereas port isolation is at the level of around − 20 dB or better for the OFF mode, and around − 30 dB for the ON mode, both for side-by-side and face-to-face orientation.

Numerous dynamically frequency-reconfigurable antennas have been reported in the literature. Table 2 compares the proposed frequency-reconfigurable R-TSM antenna and the representative state-of-the-art designs. As observed, the benchmark designs are either limited to the UWB band^42–44^, exhibit narrowband characteristics^43,44^, or offer frequency tunability only at specific resonant frequencies within their respective bands^36,45^. For instance, Shuriji et al*.*^45^ introduced a 5-mode frequency-tunable microstrip antenna utilizing two PIN diodes and two capacitors, achieving resonant frequencies across the S-band at 2, 2.09, 2.18, 2.27, and 2.4 GHz, the C-band at 4.9, 7.7, and 7.9 GHz, the X-band at 10.5 and 10.7 GHz, and the Ku-band at 13.3, 13.4, 14.9, 15.2, 15.5, 15.6, 17.4, 17.5, and 17.6 GHz. Nonetheless, the mentioned design lacks the capability to dynamically switch frequencies between the S, C, X, and Ku bands^45^. Rao et al*.* presented a frequency-reconfigurable design that operates between the Ku and K bands^46^. Salamin et al*.* proposed a wideband/band-notched antenna design, with the first mode operating over a wideband antenna with a range of 4.4–14.7 GHz and the second mode operating between 2.9 GHz and 14.3 GHz with dual notched bands at 3.6 and 5.6 GHz. This design eliminates interference from WiMAX, C-band satellite (downlink) systems, and DSRC bands^47^. However, it also lacks the capability to dynamically switch between the UWB and Ku band. The proposed design has the capability to dynamically switch frequencies between the UWB region, including the ISM band (5.8 GHz), WLAN band (5.2 GHz), lower X-band (8 GHz), and Ku band (12.7–15.65 GHz). As mentioned earlier, the gain of the proposed antenna is not as high as gain of some of the benchmark devices. However, this is an inherent property of monopole antennas associated with their omnidirectional radiation patterns, prioritizing wide coverage rather than high gain. This property makes the antenna suitable for diverse broadband applications.

**Table 2:** Comparison of the proposed R-TSM antenna with related designs.

Ref	Size (mm^2^)	Substrate	No. of modes	Reconfigur. bands	Resonance freq. (GHz)	Bandwidth per bands (GHz)	Gain (dBi)	Reconfigur. type
^42^	27 × 25	RO4350B	4	LTE	2.3/4.5	2.29–2.39/4.40–4.52	–	PIN Diodes
				AMT	4.5 / 5.8	4.29–4.57/5.71–5.93		
				WLAN	2.3	2.3–2.4		
					5.8	5.64–5.96		
^43^	20 × 10	RO4350B	2	C-Band	6.8	–	-4.4	S-PIN Diodes
				Ku-Band	15.5	–	-3.5	
^44^	53.5 × 53.5	RT5880	2	S-Band	2.417	0.018	2.37	PIN Diodes
				C-Band	5.84	0.322	6.74	
^45^	31.1 × 27	FR-4	4	S/C/Ku	2.18/5.51/14.96/17.48	–	7.3	PIN Diodes Capacitor
				S/C/X/Ku	2/7.76/10.5/13.3/15.5/17.57			
				S/C/X/Ku	2.09/4.9/7.7/10.7/13.3–17.6			
				S/C/Ku	2.27/4.97/7.94/14.96/17.48			
^46^	3.6 × 5.7	FR-4	2	Ku-Band	14.9	0.8	3.5	PIN
				K-Band	25.5	1.4	2.7	Diodes
^47^	26 × 25	FR-4	2	WLAN/X	5.6	4.4–14.7	6.5	PIN Diodes
				UWB/Ku	3/4.8/6.2	2.9–3.2/4.3–5/6–14.3		
This work	20 × 30	FR-4	2	UWB	3.16/5.6	2.68–8.55	2.36	PIN Diodes
				Ku-Band	13.7	12.7–15.65	3.85	

## Conclusion

This study presented a microstrip-fed frequency reconfigurable R-TSM antenna with a defected ground structure for UWB/Ku band applications. The etching of rectangular slits tilted at 15 degrees on the patch surface has effectively enhanced the bandwidth, while additional slots in the ground plane improved the impedance matching. Upon optimization, the antenna dimensions are finalized at 20 × 30 mm. A transition between these bands is accomplished to achieve dynamic frequency reconfigurability, rather than restricting the antenna to operate in a fixed band (UWB or Ku band). This is done by controlling the antenna’s electrical length through circular rings placed near the feed line, alongside managing capacitive and inductive effects using PIN diodes. The antenna demonstrates dual band switching operation, covering frequency ranges of 2.68–8.55 GHz and 12.7–15.65 GHz, contingent on the on/off state of the PIN diodes. In the OFF state, the antenna operates in the UWB region, covering the ISM band, WLAN, and the lower X-band, with an impedance bandwidth of –10 dB and a maximum gain of 2.36 dBi. When switched to the ON state, the antenna operates in the Ku band, achieving a gain between 2.63 and 3.85 dBi. The antenna’s ability to dynamically switch between UWB and Ku-band frequencies makes it a strong candidate for various advanced applications, including satellite communications, health monitoring, 5G networks, aerospace systems, and remote sensing technologies.

## Data Availability

All data has been included in the study.
